# Two Cases of Successful Treatment With Hachimijiogan for Irregular Menstruation

**DOI:** 10.3389/fnut.2020.00146

**Published:** 2020-09-11

**Authors:** Tatsuji Hirabayashi

**Affiliations:** Tokorozawa Akitsu Clinic, Saitama, Japan

**Keywords:** Hachimijiogan, Kampo prescription medication, crude drug preparation, case report, irregular menstruation

## Abstract

Hachimijiogan (HJG), a Kampo prescription medication composed of eight crude drugs, has been used for treatment of climacteric disturbance and irregular menstruation. In the Japanese pharmaceutical market, HJG pills consisting of a powdered mixture of these crude drugs are available, as well as a water extract preparation. In this study, two cases of successful treatment with HJG pills are reported. Case 1 was a 37-years-old woman who had irregular menstruation that had previously been treated with HJG extract granules for 3 months; however, her symptoms were not improved. Subsequent treatment with HJG pills at a dose of 40 pills/day for 10 months led to slight improvement of her menstrual cycle, and at a dose of 60 pills/day, her menstrual cycle was normalized. Case 2 was a 29-years-old woman who had irregular menstruation for more than 5 years and was previously treated with HJG extract granules, which led to slight improvement of her symptoms. Her menstrual cycle improved slightly after 9-months treatment with HJG pills at a dose of 40 pills/day and was normalized at a dose of 60 pills/day. This study suggests that the HJG crude drug preparation is more effective than the HJG extract preparation in some cases.

## Introduction

Hachimijiogan (HJG) is described in the Chinese classical medical book “Jin Gui Yao Lue” (Kinkiyouryaku in Japanese), and it is known as one of the most useful Kampo medications in Japan. HJG granules made from water-based extract preparations are widely used; however, tablets (HJG pills) consisting of a mixture of the powdered crude drug constituents are also commercially available. This study reports two cases of patients treated with HJG pills after providing written consent.

## Case One

**Figure 1:**
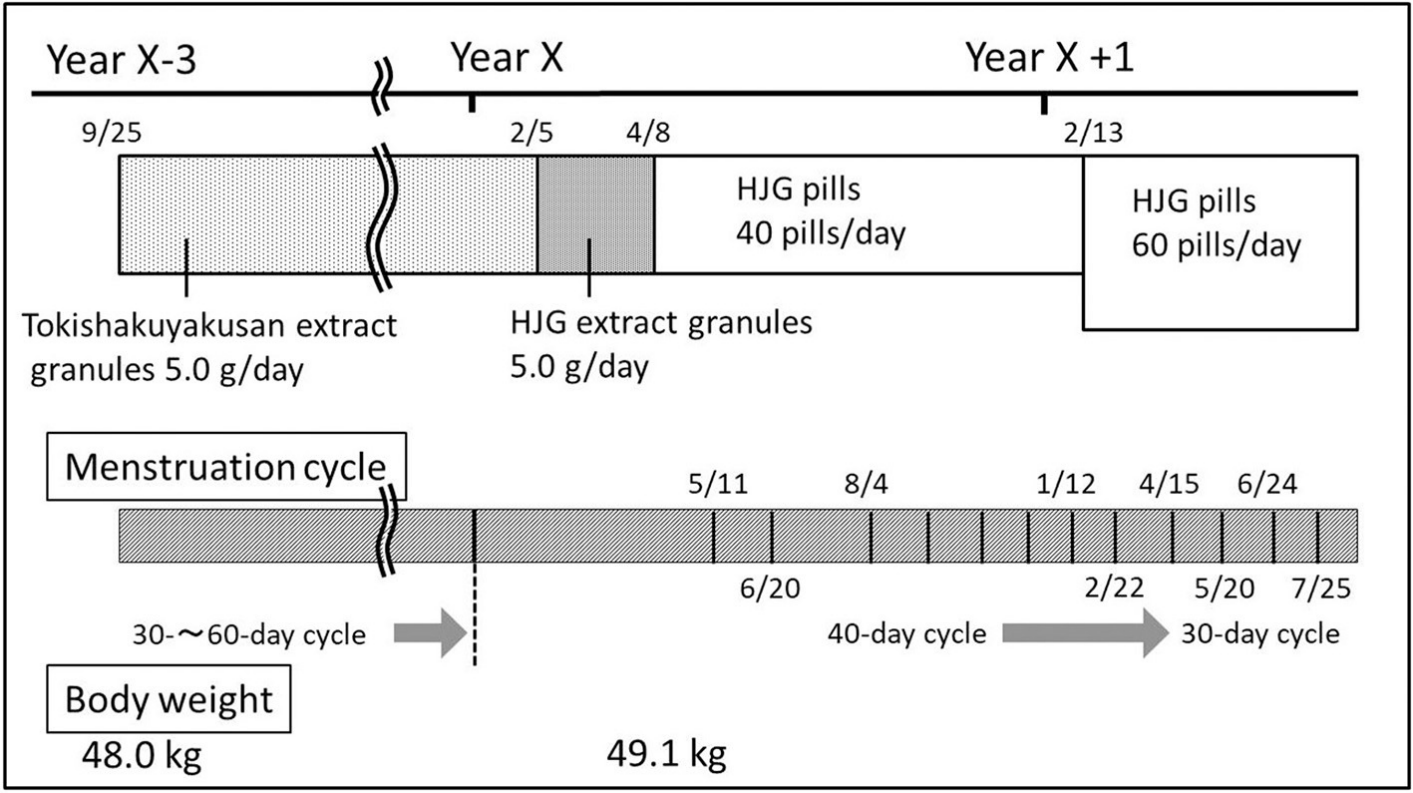
Case 1. Progress chart.

Patient 1 was a 37-years-old single female (height, 155 cm; weight, 49.1 kg; blood pressure, 108/48 mmHg) with chief complaints of cold sensitivity, tinnitus, dizziness, headache, and irregular menstruation. The patient had no noteworthy family medical history or genetic information. She had no yellowing of the conjunctiva bulbi; no conjunctival anemia; no cervical, thoracic, or abdominal abnormalities; and no edema on the anterior tibia. Considering that the patient was somewhat ectomorphic and tended to experience worsening of symptoms during menstruation, tokishakuyakusan extract granules (5.0 g/day), which are effective for treating cold sensitivity and headaches, were prescribed (Figure 1). After taking tokishakuyakusan extract granules, symptoms including cold sensitivity, tinnitus, dizziness, and headache were alleviated; however, her menstrual cycle had remained irregular. Three years after the initiation of the Kampo treatment she had no menstruation for more than 2 months, so Kampo treatment was started anew for the irregular menstruation.

Subjective symptoms included cold sensitivity of the lower body, tinnitus, and lower back pain, in addition to irregular menstruation; thus, the prescription was changed from tokishakuyakusan extract granules to HJG extract granules (5.0 g/day). Two months later, she reported no menstruation despite continuous administration of HJG extract granules.

The patient's chief complaint was irregular menstruation with subjective symptoms including cold sensitivity of the lower body and lower back pain. In addition, she was somewhat thin and had weakness of the lower abdominal region. Thus, treatment with HJG was expected to be effective for this patient, and as a result, treatment was changed to HJG pills (40 pills/day) instead of simply increasing the dose of HJG extract granules.

One month after the administration of HJG pills, the patient had menstruation for 5 days. She continued taking the medication and menstruated again, but her cycle was abnormally long at around 40 days. Hence, the dose of HJG pills was increased from 40 to 60 pills/day. Since then, her menstrual cycle has become ~30 days.

## Case Two

Patient 2 was a 29-years-old single female pharmacist working at a hospital (height, 158 cm; weight, 48.0 kg; blood pressure, 102/54 mmHg) with menstrual irregularities. The patient had no noteworthy family medical history or genetic background. She had no yellowing of the conjunctiva bulbi; no conjunctival anemia; no abnormalities in the neck, chest, or abdomen; and no edema on the anterior tibia. Her subjective symptoms were as follows; slightly dark skin, somewhat ectomorphic, normal food intake, no sleep disorder, no cold or heat sensitivity, no chills, no headache, no shoulder tension, no dizziness, lower back pain due to work in a standing position, tendency to have swelling of the lower limbs, no urination during the night, and defecation once per day.

**Figure 2:**
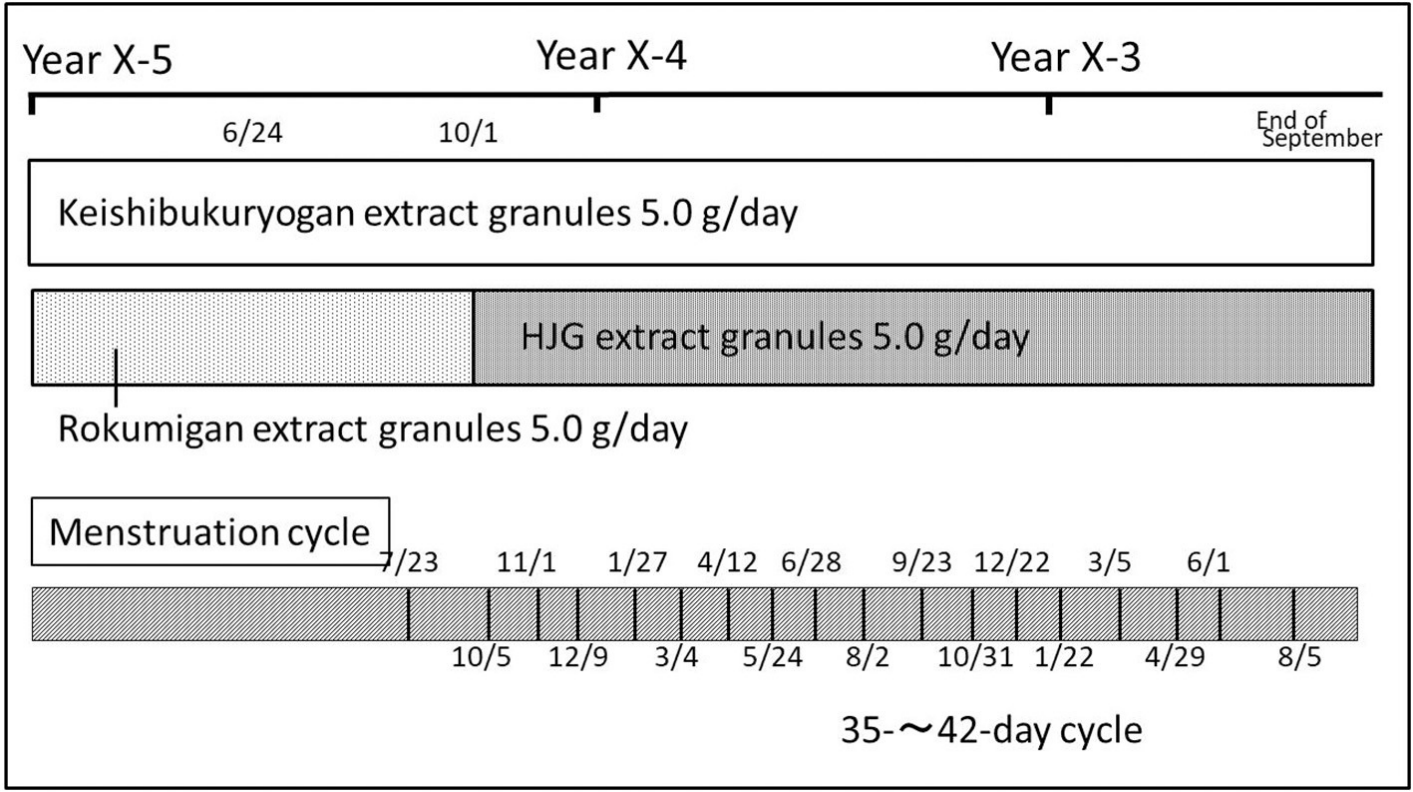
Case 2. Progress chart 1.

The patient had her first menstruation at age 12, and had a menstruation cycle of 35–42 days until the age of 18. Since the age of 19, her menstrual cycle became longer, and her menstruation stopped briefly. She visited a gynecologist and found out that her basal body temperature was irregular rather than biphasic. Hence, she underwent hormone therapy. She continued the treatment on a regular basis and maintained a normal menstrual cycle; however, she experienced an absence of menstruation whenever the treatment was interrupted. Treatment with Kampo medicine had been considered for the improvement of her symptoms, and she visited the medical institution previously indicated, where she was prescribed and started treatment with 5.0 g/day keishibukuryogan extract granules and 5.0 g/day rokumigan extract granules (Figure 2).

She first visited our hospital with the same chief complaint. At first, the prescription from her previous doctor was continued, but menstruation was irregular: therefore, she was switched from 5.0 g/day rokumigan extract granules to 5.0 g/day HJG extract granules to normalize her unstable menstrual cycle. She continued taking the prescribed drug for roughly 2 years, during which her menstruation was on a 35–42-days cycle without hormonal therapy. Although the cycle was relatively long, she considered that the cycle had stabilized and stopped taking the extract granules at her own discretion. After ceasing to take the drug, her menstruation cycle remained the same for 3 years. Subsequently, she had not menstruated for the past 2 months: therefore, she visited our hospital.

**Figure 3:**
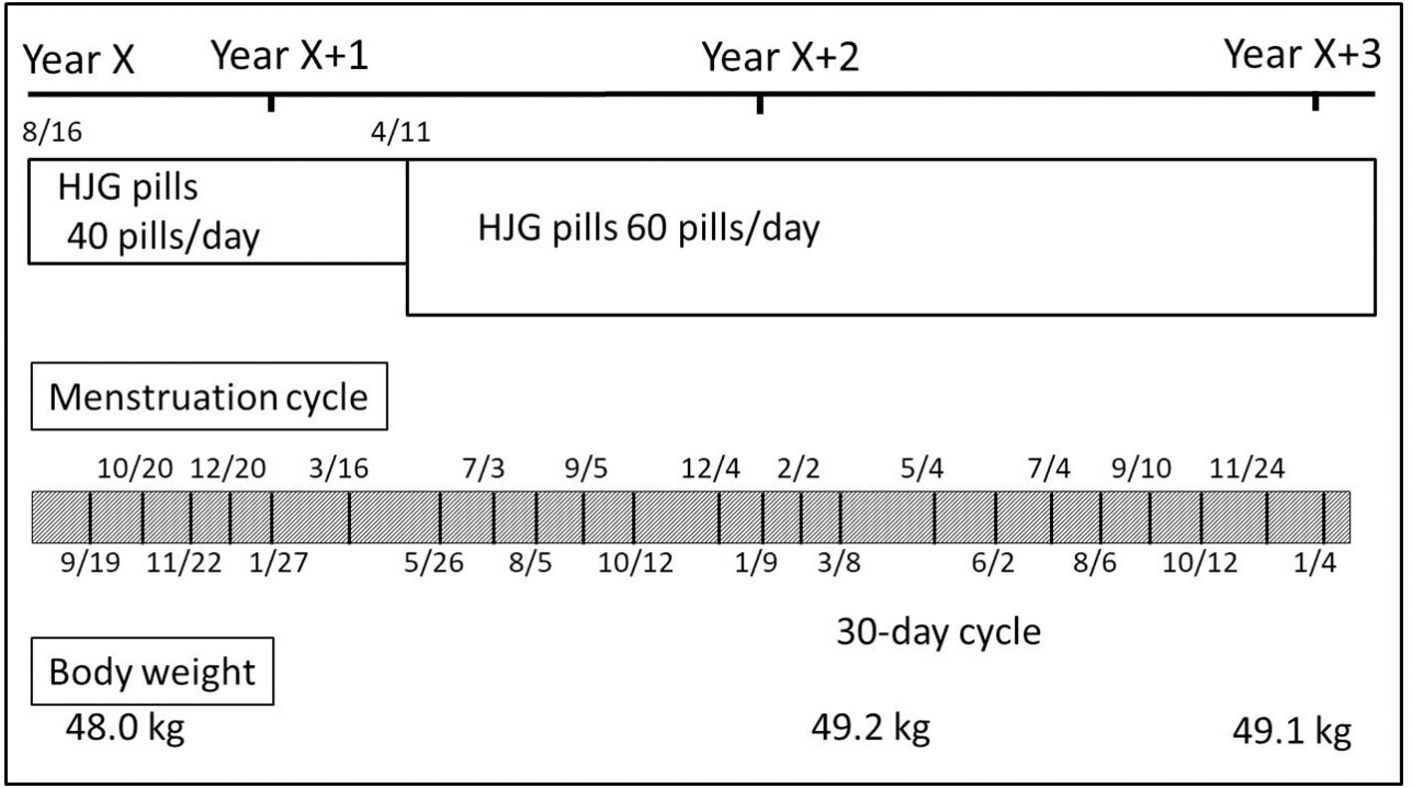
Case 2. Progress chart 2.

Her symptoms were lower back pain related to work and swelling of the lower limbs without any major complaints other than the chief complaint of menstrual irregularity. She had been taking keishibukuryogan extract granules and HJG extract granules until a few years prior, observing that it positively affected her menstrual cycle. Thus, HJG was expected to be effective considering that she had only a few subjective symptoms of lower back pain and swelling of the lower limbs. However, because her menstrual cycle was longer and she showed a benefit at the previous dose of the extract granules, treatment was initially started with HJG pills (40 pills/day) (Figure 3). Approximately 1 month after the initial administration of the pills, she menstruated for 5 days. Subsequently, her menstrual cycle became more regular at ~1 month intervals. However, 5 months after the initial administration of the pills, she started to have a somewhat longer menstrual cycle and missed her menstrual period for 49 days during the subsequent period. Thus, the dose of HJG pills was subsequently increased from 40 to 60 pills/day. Consequently, she started having a regular menstrual cycle (e.g., 34 days after and 32 days after). Since then, she has had regular menstruation occurring in nearly 30-days cycles.

## Discussion

HJG is composed of eight crude drug powders: Rehmanniae Radix, Corni Fructus, Dioscoreae Rhizoma, Alismatis Tuber, PORIA, Moutan Cortex, Cinnamomi Cortex and Aconiti Radix, and is a Kampo medication used for the treatment of the diseases referred to in Kampo medicine as being characterized by reduced function of tissues that control growth, development, and overall fertility (1). HJG has been found to be effective for many diseases and symptoms including diabetes, hypertension, back pain, edema, nephritis, bronchial asthma and dementia, and many successful cases of its use have been reported (2–5).

In particular, there have been many reports of using HJG for infertility. In a study by Usuki et al. oral administration of HJG granules (5.0–10.0 g/day) in 27 patients, aged 24–38 years, with hyperprolactinemia infertility improved their blood prolactin levels and led 12 cases (44%) to pregnancy (6).

Furthermore, Shima et al. reported that administration of HJG extract granules (7.5 g/day) or HJG pills (60 pills/day) in 50 intractable infertility cases resulted in pregnancy in a total of 45 cases (90%) within 6 months (7). Among the cases in which pregnancy was not possible during treatment with HJG extract granules, there were many cases in which pregnancy could be achieved by switching the prescription to HJG pills. With respect to gynecologic diseases, there have been many reports on tokishakuyakusan, keishibukuryogan, unkeito, kamishoyosan, and other Kampo medications. In contrast, there have been relatively few reports on the use of HJG for gynecologic diseases. One of the reasons could be that HJG contains aconite root, which is used to relieve pain in people with weak constitutions, often including “after middle age,” “pain,” and “sharp pain” in the descriptions. Thus, there has been reluctance to use it in young women.

The two cases in the present study had reported experiencing menstrual irregularity observed for a relatively long time. In the first case, there was no significant effect on the menstrual cycle after several years of taking tokishakuyakusan extract granules, which are frequently used to treat irregular menstruation. As a result, she was switched to HJG, taking into account her symptoms of cold sensitivity in the lower back and lower limbs, and lower back pain. She experienced no improvement during treatment with extract granules, while her symptoms improved after switching to the pill formulation.

The second case had a history of taking both rokumigan extract granules and HJG extract granules, and the extract granules were also effective. However, considering her back pain and edema of the lower limbs, which often occurred in the evening, she was treated with the crude drug HJG alone instead of the extract granules. In both cases, a dose-dependent effect was observed. Both patients were satisfied with the treatment due to the improved menstrual cycle.

A study by Toriizuka et al. compared the difference in effect between keishibukuryogan as a decoction and in pill form with regard to (8) and a clear difference in their medicinal effects was reported (9). They noted that the constituents of keishibukuryogan decoctions and pills differed and hypothesized that the differences may have occurred because the extraction efficiency of the essential oil component is poor in the water-based extract preparation, while the pill contains all the components as a solidified crude drug powder.

Hachimijiogan contains essential oils such as paeonol and paeoniflorin, a fat-soluble ingredients, which are considered to be effective in improving blood flow (10).

Since pills are made by powdered crude herbs without the process of extracting, both water-soluble and fat-soluble ingredients are contained. On the other hand, the extract granules are mainly composed by water-soluble ingredients. Based on this fact, pills might contain a large amount of essential oil ingredients such as paeonol and paeoniflorin due to the difference in manufacturing method compared to the extract granules. In our cases, the reason why the menstrual cycle was improved might be that the fat-soluble ingredients composed in the pills improved the disturbance of blood flow due to pelvic congestion.

Regarding studies on HJG, Shima et al. reported that the use of powdered crude drug tablets improved the effect of infertility treatment, as described above. Similarly, the cases in this study indicate that the crude drug preparation had enhanced efficacy compared with the extract preparation. However, this study is an inference based on two case reports, and it is necessary to consider many cases in the future.

## Translation

This paper was submitted to a journal written in Japanese (philkampo No. 55, 25–27, 2015). It was rewritten in English with permission from the magazine so as to reach a global audience.

## Data Availability Statement

The original contributions presented in the study are included in the article/supplementary material, further inquiries can be directed to the corresponding author/s.

## Ethics Statement

Written informed consent was obtained from the individuals for the publication of any potentially identifiable data included in this article.

## Author Contributions

The author confirms being the sole contributor of this work and has approved it for publication.

## Conflict of Interest

The author declares that the research was conducted in the absence of any commercial or financial relationships that could be construed as a potential conflict of interest.
